# Electro Physiology

**Published:** 1845-12-01

**Authors:** 


*Electro-Physiology.*—The Medico-Chirurgical Review for April, 1845, contains an interesting article on the above subject. The article is chiefly an analysis of two late (untranslated, we presume,) French works by M. M. Longet, and Ch. Matteucci. The results of the experiments made by these gentlemen seem to us curious and important. We cannot, however, transfer the details to our columns, but must refer to the article alluded to for these. We were particularly interested in the fact that the *motor*, *sensitive* and *mixed* nerves are capable of being distinguished, and their separate properties demonstrated, after death, by means of the electrical current. This method of illustration will, therefore, we hope, supersede the cruel experiments which have been hitherto, especially in France, practiced, without remorse, on living animals. These gentlemen have ascertained, that when the sensitive or posterior roots are separated from the spinal cord, no muscular contractions can be excited by the current, whatever be its direction—thus showing that they are destitute of any motor properties. They have, also, shown that the posterior columns of the cord are entirely devoid of a motor function, while the anterior are exclusively motor.

With reference to the therapeutic uses of the electric current, the deductions which they draw from their researches are as follows: Paralysis either of motion, or sensibility, or both, constitute the maladies for which it especially deserves to be tried. They also propose it as a remedy for tetanus, and give one case in which it produced a temporary alleviation. The rationale of its operation in the latter affection, seems to consist in the production of paralysis by powerful shocks, or an uninterrupted current, and, in this way, the removal of the tetanic condition of the muscles.

In its application, two electro-physiological facts are to be kept in mind. The first is, that an electric current, if transmitted through a nerve for a certain period of time, destroys the sensibility of the nerve, or, in other words, paralyzes it. If allowed to remain in repose, the nerve, after a certain interval, recovers its excitability. But it has been ascertained by Matteucci that the excitability may be restored in a much shorter period by passing a second current through the nerve in the reverse direction. The second fact to be borne in mind is, that if the nerves of a living animal be submitted to the passage of the electric current, repeated at short intervals, tetanic contractions are excited; and if the experiment be continued for some time, the nerves entirely loose their excitability."

"These are the facts," says Matteucci, "which, independently of all theory or hypothesis, should guide us in the therapeutical application of the electrical current to palsies. We have seen that to restore the excitability to a nerve which had been deprived of it, by an electric current, it is requisite to conduct the current in an opposite direction. Hence, to cure the paralysis, the current should be passed in a contrary direction to that which has produced it. In a paralysis of motion, the inverse—centripetal—current should be employed; while in paralysis of sensation,, the direct—centrifugal—current should be used. In paralysis both of motion and sensation, there is no reason to induce us to prefer one current to the other.

Theory, also, teaches us a rule in its application: never to continue the current too long, lest we augment the disease we wish to cure. The more intense the current, the shorter should be its duration; and as we have seen that the passage of the electric current in the nerves repeated at short intervals of time considerably enfeebles their sensibility when continued for a long time, we must take care and not pass from one extreme to another. Theory advises us to apply the electric current of an intensity which should vary with the degree of the malady, and continue its passage for two or three minutes, at intervals of some seconds. After these two or three minutes, during which we shall have communicated some twenty to thirty shocks, we should leave the patient at rest for some time, and then renew the treatment."

